# Genome-scale MicroRNA target prediction through clustering with Dirichlet process mixture model

**DOI:** 10.1186/s12864-018-5029-7

**Published:** 2018-09-24

**Authors:** Zeynep Hakguder, Jiang Shu, Chunxiao Liao, Kaiyue Pan, Juan Cui

**Affiliations:** 1 Systems Biology and Biomedical Informatics (SBBI) Laboratory, Department of Computer Science and Engineering, University of Nebraska-Lincoln, Lincoln, NE 68588 USA; 2 Department of Electrical and Computer Engineering, McGill University, Quebec, Canada

**Keywords:** MicroRNA, MicroRNA target prediction, Dirichlet process Gaussian mixture, Machine learning, Bayesian inference, Dynamic microRNA regulation

## Abstract

**Background:**

MicroRNA regulation is fundamentally responsible for fine-tuning the whole gene network in human and has been implicated in most physiological and pathological conditions. Studying regulatory impact of microRNA on various cellular and disease processes has resulted in numerous computational tools that investigate microRNA-mRNA interactions through the prediction of static binding site highly dependent on sequence pairing. However, what hindered the practical use of such target prediction is the interplay between competing and cooperative microRNA binding that complicates the whole regulatory process exceptionally.

**Results:**

We developed a new method for improved microRNA target prediction based on Dirichlet Process Gaussian Mixture Model (DPGMM) using a large collection of molecular features associated with microRNA, mRNA, and the interaction sites. Multiple validations based on microRNA-mRNA interactions reported in recent large-scale sequencing analyses and a screening test on the entire human transcriptome show that our model outperformed several state-of-the-art tools in terms of promising predictive power on binding sites specific to transcript isoforms with reduced false positive prediction. Last, we illustrated the use of predicted targets in constructing conditional microRNA-mediated gene regulation networks in human cancer.

**Conclusion:**

The probability-based binding site prediction provides not only a useful tool for differentiating microRNA targets according to the estimated binding potential but also a capability highly important for exploring dynamic regulation where binding competition is involved.

**Electronic supplementary material:**

The online version of this article (10.1186/s12864-018-5029-7) contains supplementary material, which is available to authorized users.

## Background

MicroRNAs (miRNAs) are important post-transcriptional gene regulators that silence messenger RNA (mRNA) targets via mRNA degradation or translational repression [1, 2]. They hybridize with complementary sequences in the 3′-untranslated regions of mRNA, particularly in the “seed region” (*2nd-8th* bases on the 5′ end), for their binding [3]. In RNA-induced silencing complex, both miRNA and mRNA are degraded if the miRNA nucleotide sequence has a high degree of complementarity to the sequence in the mRNA target [4, 5]; otherwise, the binding of miRNA to mRNA will halt mRNA translation without causing degradation [5, 6]. The large-scale miRNA-mRNA interactions detected by sequencing analyses has shown various interaction patterns, e.g., many interactions happen via complementary sequences in discontinuous regions other than seed region [7], indicating different regulatory mechanisms. In addition, compelling evidence reveals the dynamic nature of miRNA-mRNA interaction that multiple miRNAs can bind to the same mRNA sequence or different copies of the same transcript -- cooperative interactions [8], while multiple different mRNAs, possibly also with other types of RNA, e.g., long non-coding RNAs and circular RNAs [9], can compete for binding to the same miRNA -- competitive interactions [10]. Furthermore, other factors, such as genetic mutations [11–14], competition with RNA binding proteins [15, 16], and conditional expression of miRNA and mRNA can also affect the status of miRNA-mRNA interactions. All listed above indicate the complexity underlying miRNA-mediated gene regulation.

The molecular mechanisms have been partially clarified by extensive studies focusing on miRNA biogenesis and function [17, 18], which also show the participation of miRNAs in virtually every aspect of cellular activities starting with differentiation and development of cells. MiRNAs affect normal functioning of the cell including metabolism, proliferation and apoptotic cell death as well as malfunctions such as viral infection, and tumorigenesis [19–24]. In humans, it is estimated that 2500+ miRNAs can regulate over 60% of human genes [25]. Research interest in miRNA regulation has been dramatically increasing, resulting in numerous computational tools such as TargetScan [26], miRDB [27], miRanda [28], and mirSVR [29]. The miRNA targets predicted by these tools can be used to indirectly infer miRNA function, e.g., through pathway enrichment analysis [23]. However, the complexity of miRNA-mRNA binding, especially the cooperative and competitive binding modes observed with miRNAs complicates the target prediction task. Most methods that focus primarily on finding complementary sequences in the seed region fail to address this complexity.

Despite the challenges faced in computational prediction, novel sequencing techniques has facilitated experimental discovery of a large number of miRNA-mRNA interactions. For example, the crosslinking, ligation, and sequencing of hybrids (CLASH) analysis has identified 18,514 miRNA-mRNA interactions where 60% of the interactions were associated with seed region [7]. Further, the coding regions of mRNAs were shown to house ~ 60% of the binding sites. The existing algorithms are designed on the assumption of 3’-UTR centric binding, new algorithms will need to revise this assumption. Another study using covalent ligation of endogenous Argonaute–bound RNAs (CLEAR)-CLIP in human hepatoma cells corroborated the above results: ~ 26% of the interactions were associated with seed region and ~ 57% are non-3’UTR interactions [30].

In this study, we designed a new computational method for miRNA target prediction using Dirichlet Process Gaussian Mixture Model (DPGMM) [31], with integration of the large-scale sequencing-detected interactions. The main aim is to infer interactions along with indicated confidence. Given the large number of interaction patterns miRNAs and mRNAs can have (to-be-discovered) and the uncertainty about source of similarity, clustering is the tool of choice to group similar interactions with respect to either the miRNA or mRNA involved. In clustering tasks where the number of clusters are not known ahead of observing the data, the non-parametric Bayesian method DPGMM is commonly used [32–36]. DPGMM also has advantages in accommodating clusters with various sizes and structures, free specification of the number of clusters, easy computation, and interpretability [20], compared to other multi-class learning systems, such as SVM, K-means, and GMM clustering. To accomplish, we first considered a large number of molecular features related to miRNA-mRNA binding sites including sequence pairing [26], evolutionary conservation [37], free energy of the miRNA-mRNA heteroduplex [38], target site accessibility [39], and the flanking sequence of the target site on mRNA. A few novel features possibly associated with binding efficacy were also considered, such as AU-rich nucleotide composition near the binding site, proximity to sites for co-expressed miRNAs (possibly associated with cooperative action), proximity to residues pairing at miRNA nucleotides 13–16, positioning away from the center of long UTRs [2, 40]. In addition, several statistics related to binding site were also assessed, e.g., the number of complementary pairs within seed region and/or within the whole biding site. Based on these heterogeneous features, a feature vector will be constructed for each given miRNA-mRNA interaction, as input to the model.

For each candidate interaction, the new system can output an assignment score as posterior probability for each of the clusters. By assessing all interactions from the same cluster, one can explore new insights in interactive patterns reflected by each identified cluster. In addition, based on the sequence information of experimentally-detected interactions and aforementioned distinguishing features, this system will allow one to assess if one miRNA can bind to a specific splicing transcript, a very unique feature highly useful in practice when compared to gene-level prediction offered by existing tools. At last, we demonstrated in a breast cancer case study the application of predicted target information to infer conditional miRNA-gene interaction through modeling dynamic gene regulation while considering multiple other gene expression regulation mechanisms such as transcription factor and copy number variation (CNV).

## Methods

### Data preparation and feature generation

**Fig. 1:**
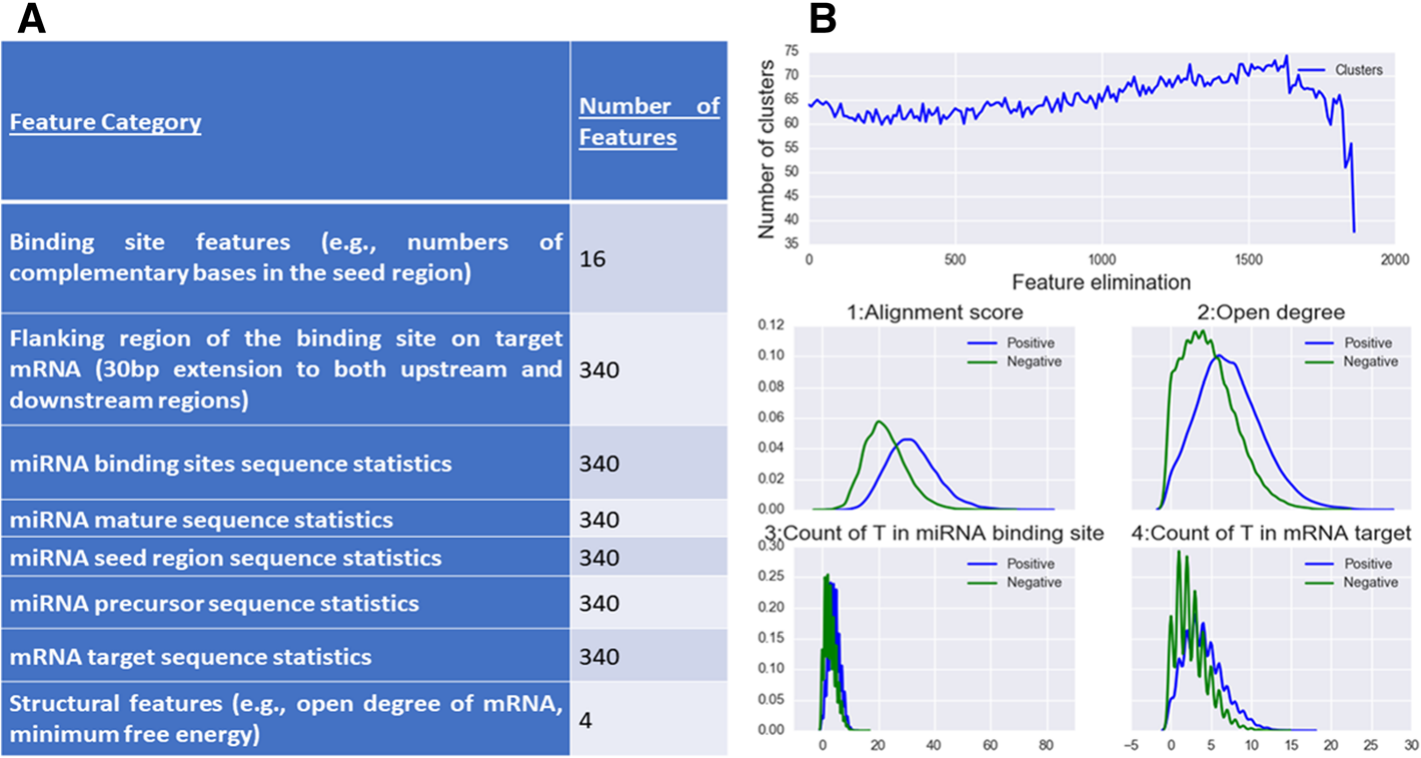
Interaction features used in the model. (**a**) Breakdown of features according to categories. (**b**) Recursive feature elimination process. Top panel shows the performance of the feature elimination process on the initial set of 2059 features. Bottom panel illustrates the distributions of four discriminative features in the positive and negative datasets

Table 1 summarizes the miRNA-mRNA interactions used in this study. Experimentally identified interactions collected from public databases and interactions reported in sequencing analyses constitute the training and validation data [41]. For each interaction, an initial set of 2059 features were generated. Besides general structure and sequence features reported in the literature [2, 40] [3, 26], we explored new features such as the length of an interaction and the flanking sequence of the binding site on mRNA. We included the frequencies of all possible *k*-nucleotide combinations (*k* = 1,…,4) on both miRNA and mRNA sequence involved in an interaction. RNAFold was used to calculate the minimum free energy, secondary structure, and open degree of each binding site [3]. A summary of all features compiled in this study is given in Fig. 1A, with details provided in Additional file 1.

**Table 1:** Datasets applied in this study

Datasets	Content
CLASH data [7]	17,436 interactions on Human kidney cell (HEK293), associated with Ago1
iPAR-CLIP data [8]	10,566 interactions on HEK293, human embryonic stem cell, EBV-infected lymphoblastoid cell lines, and primary effusion lymphoma cell line, associated with Ago1 and Ago2
CLEAR-CLIP data [30]	32,711 interactions on Human hepatoma cell, associated with Ago
mirTarbase [20]	11,002 interactions on Human genome, predicted by miRanda; 483 validated interactions by non-sequencing analysis
RefSeq [42]	56,000 human transcripts

### Classification using mixture model

Positive training data for the classifier consisted of experimentally identified miRNA-mRNA interactions. Negative training data was generated to represent miRNA-mRNA pairs that don’t interact. We summarize the whole procedures as follows.

1. *Negative Data Generation*

Negative sample generation was done by sliding a k-mer window (k = 22) across all known mRNA sequences. The commonly-used negative interaction set consisting of a small number of randomly generated interactions is often biased and not sufficient to represent the entire negative space. To address this problem, we generated a 4-tiered negative set with each tier corresponding to a different level of negative potential. Higher levels of confidence are captured in higher tiers (Table 2). For instance, level 1 data includes randomly-generated false binding sites among reported miRNA and mRNA pairs, whereas level 4 data represents interactions randomly synthesized from unreported miRNAs and mRNAs. There are infinitely many possible negative interactions between all human miRNAs and mRNAs; in order to keep the size of the negative set comparable with the positive set, we selected ~ 8000 interactions from each of the four categories randomly as representative interactions of that category.

2) Building the Classifier

**Table 2:** The positive and negative datasets

Dataset	Statistics	
	miRNA/mRNA	interaction
Pos-1: Interactions reported in CLASH data	399 7000	17,436
Pos-2: Interactions reported in iPAR-CLIP data	291 4043	10,567
Neg-1: Interactions generated on reported miRNA and mRNA pairs	755 9179	8768
Neg-2: Interactions generated on reported miRNAs and unreported mRNAs	755 20,516	8768
Neg-3: Interactions generated on unreported miRNAs and reported mRNAs	1833 9179	7332
Neg-4: Interactions generated on unreported miRNAs and unreported mRNAs	1833 20,516	7332

In order to discover unknown binding patterns and properties, a classifier was first trained to differentiate positive interactions from negative. The rationale is as follows. After feature generation, for a given miRNA *r* and a set of binding sites *I* related to *r*, the *i*th binding site is represented with an *m*-dimensional feature vector *x*_*i*_ based on a set of *m* features (m = 2059 in the initial analysis). Feature vectors corresponding to all the n sites in *I* are represented by {*x*_1_, …, *x*_*i*_, …, *x*_*n*_}. Let *n*_*c*_ be the number of clusters observed in miRNA-mRNA interactions and *z*_*i*_(*i* = 1, …, *n*_*c*_) represent the cluster membership of the interaction *x*_*i*_. We then apply Dirichlet Process Gaussian Mixture Model (DPGMM) to obtain interaction clusters. The Dirichlet Process (DP) is the prior distribution for the mixture model specifying a distribution of probability distributions. In this setting, these distributions specify the parameters of miRNA-mRNA interaction clusters. The parameters of DP are a base distribution *G*_0_ and a positive concentration parameter α. Base distribution *G*_0_ is the expected value of the process while α determines the dispersion of the distributions around *G*_0_.  A small α results in distributions that are concentrated around *G*_0_.  As α increases, the dispersion of distributions increase.

In general, in a Gaussian mixture model with *K* components, the likelihood of data is:

$$ p\left(\boldsymbol{x}|{\theta}_1,\dots, {\theta}_K\right)=\sum \limits_{j=1}^K{\pi}_j\mathcal{N}\left(\boldsymbol{x}|{\mu}_j,{S}_j\right) $$

where ***π*** denotes the mixing proportions and *θ*_*j*_ = {*π*_*j*_, *μ*_*j*_, *S*_*j*_} is the set of parameters; proportion, mean and precision, of a component in the mixture. (*μ*_*j*_, *S*_*j*_) are drawn from a distribution *G* that is in turn drawn from a DP(α, *G*_0_). Fixed covariance and a conjugate $\mathcal{N}\left(0,1\right)$ prior on the component means were used. The optimum value for α was experimentally determined to be 10 (results not shown).

In the clustering setting, we let *z*_*i*_ represent the cluster number for observation *x*_*i*_, 0 ≤ *z*_*i*_ ≤ *n*_*c*_, the prior on cluster assignments is

$$ p\left({z}_i=j\right)=\frac{n_j}{\upalpha +\mathrm{n}-1} $$

for an existing cluster *j* and

$$ p\left({z}_i=K+1\right)=\frac{\upalpha}{\upalpha +\mathrm{n}-1} $$

for a new cluster. Here, *n* is the total number of data points and *n*_*j*_ is the number of data points in cluster *j*.

Cluster assignments are done using the normalized log posteriors of clusters. The log posterior is:

$$ \log \left({n}_j\right)+\log\ \frac{\exp \left(-\frac{1}{2}{\left(x-\mu \right)}^T{\Sigma}^{-1}\left(x-\mu \right)\right)}{\sqrt{\left|2\pi \Sigma \right|}} $$

for existing clusters and

$$ \log \left(\upalpha \right)+\log \frac{\exp \left(-\frac{1}{2}{\left(x-\mu \right)}^T{\Sigma}^{-1}\left(x-\mu \right)\right)}{\sqrt{\left|2\pi \Sigma \right|}} $$

for new clusters, where x is the feature vector, μ is the cluster mean vector and 힢 is the covariance matrix for the cluster. Normalization factor is the sum of the log posteriors. A new observation is assigned to the cluster with the highest normalized posterior probability. Ties are broken randomly during cluster assignment.

We trained the DPGMM model on the training set consisting of positive and negative interactions until the system converged. 25% of the total dataset was kept out of training to be used as test data. Each interaction was assigned to the cluster which has the highest posterior probability of assignment. This model provides the flexibility of assessing the accuracy of the clustering at different levels: 1) whether a new interaction can be correctly assigned to a positive or negative cluster, or 2) whether a new interaction is assigned to a cluster that contains the participating miRNA. Several metrics have been used to evaluate the performance including the sensitivity, specificity, accuracy and Matthews correlation coefficient (MCC). Optimization of the model was done by a grid search of parameters over a large range. The main parameter of the DPGMM model is the α parameter, for which the values 10, 30, 60, 90 and 100 were considered. The model was trained for different number of iterations, namely 10, 30, 60, 90 and 100. Bayesian Information Criteria (BIC) [42] were obtained for each possible combination of the above values of the parameters. The combination that resulted in the lowest BIC were used in the final model.

3) Feature Selection

A feature elimination analysis was performed on the initial set of 2059 features to remove unrelated and noisy features and search for the minimal set of relevant features that optimize classification performance. We performed the first filtering step based on t-test on each feature between positive and negative data sets where 44 non-discriminative features with *p*-value > 0.05 were removed from our initial feature list. At this elimination step, multiple hypothesis correction methods were not applied. Next, an independent logistic regression classifier was built for each of the remaining features. Each feature was associated with the Area Under the Curve (AUC) value resulting from its corresponding logistic regression classifier and features were ranked according to their AUC values. Then we followed a recursive feature elimination (RFE) procedure to fruther remove features irrelevant or negligible to our classification goal. The procedure is as follows: a DPGMM classifier was built using the remaining features after the first elimination step and the 5-fold cross validation accuracy of the model was recorded. Next, we removed the feature with the lowest AUC value and performed DPGMM classification again. We recursively removing the least important feature and performing DPGMM classification until a minimal set of features, without losing the classification performance, is obtained. The remaining features were used for the final model.

4) Cascade Model for MiRNA Specific Clusters

**Fig. 2:**
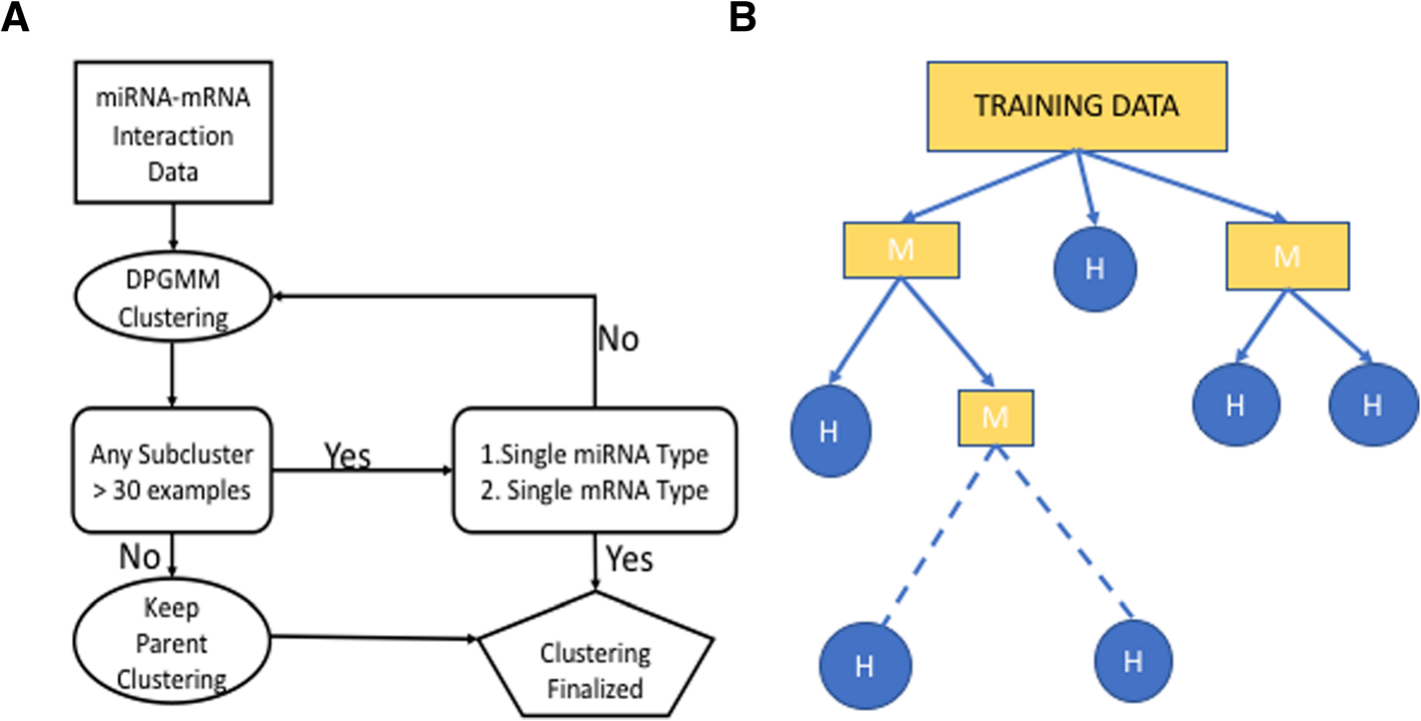
Cascade DPGMM model. (**a**) Flowchart describing training of the model. (**b**) Illustrative example of cascade DPGMM. Rectangles represent mixed clusters (M), circles represent homogeneous clusters (H). Clustering goes on until homogeneous clusters are obtained

Our model offers different levels of clustering results. Each successive level is more specialized than the previous level (Fig. 2). After the initial clustering, some clusters are expected to contain exclusively negative interactions, while others may contain both negative and positive interactions. Also, the clusters contain different types of mRNA or miRNAs. For those clusters that were heterogeneous and had more than 30 examples, we continued clustering the examples into sub-clusters with additional DPGMM rounds. Stopping criteria for clustering were: 1) homogeneity of miRNA type, 2) homogeneity of mRNA type and 3) size of clusters (Fig. 2). In case of termination due to criterion 3, the sub-clusters were excluded from the final model. We construceted a clustering tree using this recursive procedure. The root level clusters are the results of the initial clustering, and the leaf level clusters are the result of the last clustering. `represent the results of the last DPGMM clustering round, we expect to see specific clusters that are homogeneous with respect to a miRNA or mRNA.

The root level clusters can be used to evaluate if a new interaction object can be correctly predicted as a real interaction or not while the lower layers can help us to evaluate if the object can be assigned to its own cluster. A certain number of mixed clusters are expected to remain in the model corresponding to different miRNAs that follow similar binding patterns.

### Model evaluation and genome-scale target screening

In this study, CLASH and iPAR-CLIP data were used to train and test the model (75% of the interactions were used as training set and 25% as testing set). The final model was decided based on performance using the remaining features after feature elimination. For independent evaluation, duplicated interactions in CLEAR-CLIP data and mirTarBase data were removed and the remaining were used.

Next, the RefSeq data [43] were used to screen the whole human transcriptome for possible binding interactions with miRNAs. To determine if a position on mRNA is a candidate site for a given miRNA, we locally aligned the sequences of the candidate site and the miRNA. An extension of 30 nucleotides on both ends were considered while computing features for a given candidate binding site. A candidate binding site and a miRNA pair were tested for a possible interaction by assigning to a cluster within the clustering tree model. Leaf level clusters were considered as final cluster assignments.

An interaction confidence metric (IC) was used to rank the predicted interactions according to how reliable they are. IC for a candidate interaction *i* and the dominant miRNA *k* in *i’*s assigned cluster is IC_ik_ = z_i_ * c_k_, where *z*_*i*_ indicates normalized assignment probability and *c*_*k*_ is the proportion of miRNA *k* in the cluster.

Last, to demonstrate the use of predicted interactions under the context of studying dynamic gene regulation, we have performed a case study of inferring conditional miRNA regulation associated with cancer progression. Based on a set of genomic data on breast cancer from The Cancer Genome Atlas (TCGA) [44] including miRNA and mRNA expressions, CNVs, and DNA methylation profiles and a meta-Lasso regression model our group has been recently developed [45], miRNA regulation associated with different cancer stages were detected. Previously, the regression model evaluates the likelihood of interactions between a pair of miRNA and mRNA based on a regulatory score (RS) which is calculated through aggregation of binding probability and binding affinity [45]. In this study, we replace the RS score by the DPGMM-derived posterior probability, the highest assignment score. Along with other factors such as Transcription factor (TF)-gene regulatory potential, Lasso regression was utilized to identify the TFs and miRNAs that regulate a specific gene under a given condition.

## Results

An initial training of the model using the whole set of 2059 features and 75% of the CLASH and iPAR-CLIP data resulted in 34 positive and 38 negative clusters. The model was tested on the test data (the remaining 25% of CLASH and iPAR-CLIP) which resulted in 82.0% overall accuracy. Table 3 shows the promising validation results on several independent datasets.

**Table 3:** Prediction performance on the training, testing, and independent datasets

Dataset	Performance			
	Sensitivity	Specificity	Accuracy	MCC
Training 75% of CLASH, iPAR, and negative	0.78	0.86	0.82	0.64
Testing 25% of CLASH, iPAR, and negative	0.77	0.86	0.82	0.64
Validation-1 CLEAR-CLIP	0.80	–	0.80	–
Validation-2 mirTarbase (validated)	0.61	–	0.61	–
Validation-3 mirTarbase (predicted)	0.62	–	0.62	–

Our model performs decently on the majority of data sets where the interaction discovery was sequencing-based, with the exception of mirTarBase. The sensitivity for mirTarBase data is low, 61.0% for the validated interactions and 62.0% for the predicted ones from other tools, respectively. The discrepancy can be due to two possible explanations. First, CLASH and CLIP interactions were detected by genome-wise sequencing where each miRNA-mRNA pair often involves many different interactions at different sites while the mirTarBase only reports a single binding site on a gene target. Second, predicted targets in mirTarBase were less reliable compared to the experimentally validated ones.

### Selected features

A subset of the initial 2059 features (Methods) that optimized the performance of the model was selected using 5-fold cross validation. A minimal set of discriminative features were kept that optimized the model by eliminating the noisy features (Fig. 1B). Feature selection was based on minimum information loss which represents the loss of the predictive power. We observed a slight increase in the overall accuracy on mirTarBase data from 61 to 62% to 64% accuracy after the least important features were eliminated recursively (with 377 remaining features).

During feature elimination, we observed that sensitivity and specificity were complementary while the accuracy and MCC were relatively consistent. The model containing 377 features resulted in the highest accuracy and MCC (see Additional file 1). We used these features in our final model. An additional table lists the top 30 selected features, ranked by AUC value that reflects the discerning power (see Additional file 2). For example, the alignment score on the binding site, the open degree of the mRNA sequence, and ‘T’ counts in both miRNA and mRNA sequences are highly distinguishing for miRNA-mRNA interaction (Fig. 1B).

### Final classification model

The final model was trained using the selected 377 features and the optimal parameters. The performance of our method was evaluated in terms of classification performance and clustering coherence as follows.

#### Classification performance

Table 4 summarizes the prediction performance on training, testing, and three independent test datasets. Using the root level clusters, the prediction results in high accuracies for both the training set and CLEAR-CLIP test set. The prediction performance for mirTarBase data set was not as desirable. One explanation for this discrepancy may be the small size of miRTarBase compared to CLEAR-CLIP which is about two times bigger. Our resulting clustering tree had a depth of 5. As clustering depth increases, more positive clusters than negative are obtained implying specialization of clusters to miRNA or mRNA types. The proportion of positive clusters within a layer increases as the tree grows in depth. The results of the Leaf layer (Table 4) shows improvement in classification performance. Highest improvement was obtained on CLEAR-CLIP data set from 87% accuracy to 92%. Both sensitivities and specificities were improved as well as accuracy. Both the differentiation of positive and negative clusters and separation of positive interactions into different clusters according to the sequences involved is more prominent in the deeper levels. Accordingly, leaf level clusters were selected to be used for prediction.

**Table 4:** Performance based on the 1st and leaf layer clusters

Dataset	Sensitivity		Specificity		Accuracy		MCC	
	1st	Leaf	1st	Leaf	1st	Leaf	1st	Leaf
Training 75% of CLASH, iPAR, negative	0.93	0.94	0.96	0.97	0.95	0.96	0.89	0.91
Testing 25% of CLASH, iPAR, negative	0.93	0.94	0.96	0.97	0.94	0.96	0.89	0.91
Validation-1 CLEAR-CLIP	0.87	0.92	–	–	0.87	0.92	–	–
Validation-2 mirTarbase (val.)	0.63	0.60	–	–	0.63	0.60	–	–
Validation-3 mirTarbase (pre.)	0.60	0.58	–	–	0.60	0.58	–	–

#### MiRNA/mRNA specific clusters

**Fig. 3:**
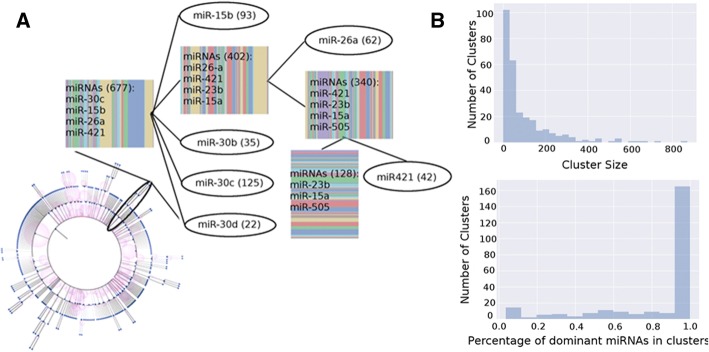
Clusters resulting from cascade DPGMM model. (**a**) Clustering tree shown at bottom left. A section of the tree is enlarged at the top right. Ellipses are homogeneous clusters. Numbers in parenthesis represent number of examples in the cluster. Striped boxes are mixed clusters, width of each stripe corresponds to the percentage of a miRNA in the mixture. (**b**) (top) the distribution of number of examples in clusters over all clusters; (bottom) distribution of percentages of dominant miRNAs in each cluster. A dominant miRNA has highest presence in a cluster

At the leaf level, the final model had 281 clusters, 244 of which were positive. We observed that 136 (56%) of the positive leaf clusters consist of only a single type of miRNA. When the homogeneity level required for each cluster (percentage of interactions associated with the same miRNA) drops, the proportion of homogeneous clusters increases, e.g., up to 73% (178 clusters) at homogeneity threshold at 80% and up to 167 (68%) clusters at homogeneity level 90%. We show an example hierarchy of clusters in Fig. 3A. At the root layer, the cluster has multiple miRNA types; miR-30c, miR-15b, miR-26a, and miR-421 constitute a high proportion of this cluster. In the subsequent layer, miR-15b and mir-30c are separated into their own clusters, as well as miR-30b and miR-30d. In the deeper layers, both miR-26a and miR-421 form their own clusters. In addition, we observed that the percentages of the dominant miRNA (miRNA with highest presence) in clusters are high (Fig. 3B).

### Validation study

Our method was compared with other state-of-the-art miRNA target prediction tools including TargetScan [26], miRDB [27], microT [46], and microT-CDS [47], based on the predicted positive interactions (Additional file 2). While our method only predicted 22,215 interactions among 550 miRNAs and 7529 mRNAs, others show significantly higher numbers that are beyond the census estimation. For example, TargetScan, microT and microT-CDS all predicted over millions of interactions among these miRNAs and mRNAs, which implies higher levels of false positives. When comparing the average targets per miRNA and average miRNA regulators per mRNA, our results are closer to reality. On the other hand, all four exisitng tools reported greater number of targets for one miRNA. We used the 37,539 positive examples in our test data to compare the sensitivity of our method with these tools. Our method achieves considerably higher sensitivity (see Additional file 2).

### Transcriptome screening

**Fig. 4:**
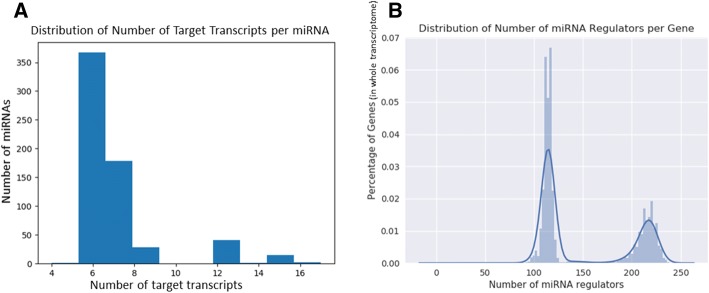
Transcriptome prediction statistics. (**a**) Distribution of number of mRNA transcript targets per miRNA. (**b**) Distribution of number of miRNA regulators per gene. y-axis shows the percentage in whole transcriptome

Clusters in our model specializes to miRNAs as cluster level becomes closer to the leaves. We leveraged this feature to not only predict if a candidate pair forms a genuine interaction but also to assess if the miRNA involved in this interaction is similar to the dominant miRNA, in terms of count, in the cluster the interaction is assigned to. The flexibility offered by our model makes screening the transcriptome at different levels possible. For example, at the lowest confidence level, where we only consider interactions with non-zero IC’s, our model predicts at least one miRNA regulator for each gene, and on average 145 miRNA regulators per gene. Figure 4 shows the distribution of number target of mRNAs per miRNA and number of miRNA regulator per gene. Considering the functional study of miRNA is largely dependent on accurate identification of its gene target, our prediction method can be highly useful as a reliable resource to facilitate downstream studies on miRNA regulation, which is also demonstrated in the next case study. The list of all predicted binding sites is available at http://sbbi-panda.unl.edu/miRCript/ (Additional online files unnecessary for review).

### A case study of conditional miRNA regulation in Cancer

**Fig. 5:**
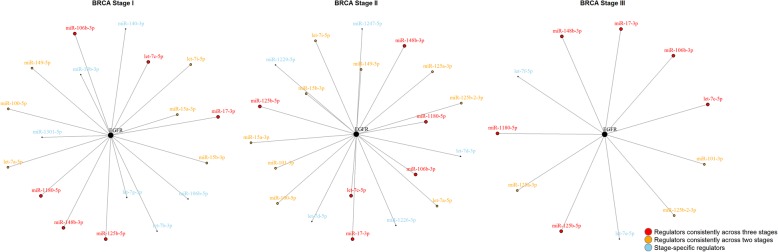
Network topologies of predicted regulators of EGFR gene in Stage I, II, and III breast cancer

Based on the aforementioned predicted miRNA target information, we have applied a regression model [45] with the DPGMM-derived assignment score representing the miRNA-mRNA regulatory likelihood. In this case, the DPGMM approach allows us to assess the binding potential among different biding sites between a gene and different miRNAs, which is important to study the competing binding among miRNAs and mRNAs. Meanwhile, it also provides a unique feature compared to other binary classification strategies. With integration of cancer associated genomic data, we were able to examine the conditional miRNA-gene regulation that are associated with tumor progression. For example, Fig. 5 illustrates the dynamic miRNA regulation pattern on gene EGFR (epidermal growth factor receptor), an important tyrosine kinase involving in cell growth and cancer development. Based on our prediction, 44 miRNAs can potentially bind to EGFR but only a subset of miRNAs play the roles under a specific condition. In the figure, we observed that different sets of miRNAs interact with the target gene across different cancer stages; some interactions are active all along during the progression (those in red) while others are stage specific (those in blue) depending on the availability of the miRNAs under different conditions. These findings are in agreement with our understanding of the dynamic regulation process.

## Discussion

Emerging sequencing technologies has changed the landscape of research on miRNA target identification. Availability of data pertaining to genome-scale miRNA interactome facilitated bioinformatics research immensely. Particularly, use of transcript-level interaction data combined with mRNA specific features in this study allows confidence in our model for transcript-level target prediction. To the best of our knowledge, splicing transcript specific miRNA binding site prediction is a novel feature that is lacking in many existing tools.

Moreover, a considerable amount of miRNA-mRNA interactions via complementary sequences have been discovered in gapped regions [11]. These findings suggest that prediction solely depending on sequence and/or contextual features such as binding energy, seed match, and conservation is not sufficient. As such the static target prediction tools that utilize only that information have received critical skepticism. We addressed this challenge in our model by incorporating data about various types of molecular features that differentiate distinct interaction patterns. Meanwhile, high false prediction miRNA target prediction rates are still a big concern.

There are several technical challenges that we encountered during the course of this work. We have used publicly available sequencing data. In compiling the data derived from different sequencing technologies, the raw sequence data needed to be reprocessed and a consolidated annotation had to be produced. Another challenge we faced is a common one in miRNA target prediction research, i.e., the negative set is huge compared to the positive set 10 times more in our case. To make our negative set more representative, we devised a new method and maintained a comparable size with the positive set.

The ambiguity around cooperative and competitive binding mechanisms adds to the complexity and semi-stochasticity associated with miRNA-mediated gene regulation. Our method can be used to infer competitive binding, since it assigns likelihoods to the numerous potential binding sites of a gene to the same miRNA which can be used to evaluate the binding potential. Our recent study shows that several miRNAs can affect a given pathway by regulating the same or different genes involved in the pathway [45]. For example, miR-18a-3p, −320a, −193b-3p, and -92b-3p co-regulate the glycolysis/gluconeogenesis and focal adhesion in cancers of kidney, liver, lung, and uterus. Similar applications shed light on miRNA regulatory mechanisms and novel roles and meanwhile, the functional studies all highlight the importance and challenges of reliable miRNA-mRNA interaction prediction.

## Conclusions

In this study we developed a new method for predicting human miRNA-mRNA interactions reliably. This statistical approach has improved prediction performance compared to similar existing tools and includes several unique features. Importantly, this tool can address practical questions such as common binding properties across miRNAs. Also, the interactions are predicted at transcript level which gives a more detailed view of interaction than the existing tools that predict gene-level binding sites. In our future work, we plan to identify miRNA co-binding module by use of conditional mRNA and miRNA genomic data, which will take the stochastic nature of miRNA-mRNA interaction into consideration. As such, we believe this study lay out the groundwork for future research on cooperative miRNA module and dynamic gene regulation.

## Additional files

Additional file 1: (Sheet 1) Whole set of features; (Sheet 2) Remaining features after elimination. (XLSX 82 kb)

Additional file 2: (Sheet 1) Top 30 selected features; (Sheet 2) Performance comparison; (Sheet 3) Sensitivity Comparison. (XLS 45 kb)
